# Neuroimaging consequences of cerebral small vessel disease in patients with obstructive sleep apnea–hypopnea syndrome

**DOI:** 10.1002/brb3.1364

**Published:** 2019-07-23

**Authors:** Shujian Huang, Dan Wang, Huiqun Zhou, Zhengnong Chen, Hui Wang, Yuehua Li, Shankai Yin

**Affiliations:** ^1^ Department of Otolaryngology-Head and Neck Surgery Shanghai Jiao Tong University Affiliated Sixth People's Hospital Shanghai China; ^2^ Department of Radiology Shanghai Jiao Tong University Affiliated Sixth People's Hospital Shanghai China

**Keywords:** cerebral small vessel disease, cognitive impairment, magnetic resonance imaging, obstructive sleep apnea–hypopnea syndrome, Virchow–Robin spaces

## Abstract

**Objective:**

This study aimed to investigate the association between severity of obstructive sleep apnea–hypopnea syndrome (OSAHS) and the neuroimaging consequences of cerebral small vessel disease (SVD).

**Methods:**

Patients with OSAHS and age‐ and gender‐matched healthy control subjects completed the mini‐mental state examination and underwent an evoked‐related potential study and overnight polysomnographic monitoring. Magnetic resonance imaging (MRI) was performed to detect markers of silent cerebral SVD, including Virchow–Robin spaces (VRS) rated on a five‐point scale, white matter lesions, lacunar infarcts, and deep microbleeds. Multinomial logistic regression models were used to examine the associations of the apnea–hypopnea index (AHI) and arousal index (AI) values, mean oxyhemoglobin saturation, the duration of snoring history, and MRI markers of small vessel disease with the incidence of enlarged VRS.

**Results:**

The study included 72 patients with severe OSAHS and 53 volunteers without OSAHS. The duration of snoring history ranged from 5 to 22 years in the OSAHS group. Smaller P3 amplitudes at Cz were found in OSAHS patients than control subjects (*p* < .05), which is associated with neurocognitive impairment. Enlarged VRS were more prevalent in the basal ganglia and centrum semiovale of patients with OSAHS than in the control group. No significant between‐group differences were observed in the number of white matter lesions, lacunar infarcts, and deep microbleeds. Enlarged VRS were positively correlated with AHI and AI values in the OSAHS group (*r* = .63, *p* < .001; *r* = .55, *p* < .001, respectively).

**Conclusions:**

Silent cerebral SVD was more prevalent in patients with OSAHS than in the controls. Enlarged VRS observed in the basal ganglia and centrum semiovale were positively correlated with severity of OSAHS, which may contribute to cognitive impairment.

## 1 INTRODUCTION

Obstructive sleep apnea–hypopnea syndrome (OSAHS) is characterized by recurrent hypoxic episodes during sleep, sleep fragmentation, and changes in sleep architecture resulting in increased cardiovascular or cerebrovascular comorbidity, and neurocognitive impairment (De Backer, 2013; Yaggi & Strohl, 2010). The progressive intermittent hypoxia and sleep fragmentation, along with changes in the cerebral blood flow, neurovascular state and neurotransmitters, the redox state of cells, and neural regulation in OSAHS patients may constitute a contributing factor to cognitive decline (Gozal, 2013; Mander et al., 2013; Poe, Walsh, & Bjorness, 2010). Sleep apnea and hypopnea are associated with substantial cerebrovascular stress (Joo, Tae, Han, Cho, & Hong, 2007; Kim et al., 2013). Furthermore, growing evidence suggests that OSAHS increases the risk of cerebrovascular events through changes in cerebral blood perfusion that affect the subcortical white matter (Alosco et al., 2013; Ramos et al., 2014). A previous study found that OSAHS was significantly associated with stroke in participants with no history of cardiovascular disease (Loke, Brown, Kwok, Niruban, & Myint, 2012). The relationship between OSAHS and cerebral small vessel disease (SVD) is complicated by the fact that the risk factors for the underlying cerebral SVD, such as hypertension, diabetes mellitus (DM), and atherosclerosis, are associated with OSAHS. Cerebral small vessel disease (SVD), with features seen on neuroimaging include recent small subcortical infarcts, lacunes, white matter hyperintensities, perivascular spaces, microbleeds, and brain atrophy, represents a major cause of vascular cognitive impairment and dementia, either by its own or in combination with neurodegenerative pathology (Pantoni, 2010). Furthermore, the relevance of correlations between OSAHS and markers of silent cerebral SVD has not been systematically evaluated. This is of particular interest because magnetic resonance imaging (MRI) studies suggest that SVD, which includes white matter lesions, lacunar infarcts, and Virchow–Robin perivascular spaces (VRS), may be a common pathogenetic mechanism underlying stroke and cognitive impairment in some brain regions (Lawrence et al., 2013). Putative links between OSAHS and cerebral vessel disease include oxidative stress, coagulopathies, and carotid atherosclerosis caused by intermittent hypoxia and sleep fragmentation (Kerner & Roose, 2016).

We used in vivo MRI to detect markers of silent cerebral SVD in OSAHS patients without hypertension, DM, or atherosclerosis and tested the hypothesis that the severity of OSAHS was associated with silent cerebral SVD which may contribute to cognitive impairment.

## 2 METHODS

### 2.1 Ethical considerations

The study was approved by the local ethics committee of Shanghai Jiao Tong University Affiliated Sixth People's Hospital; written informed consent was obtained from all subjects prior to enrollment; and the procedures followed were in accordance with institutional guidelines.

### 2.2 Study design and participants

Patients with OSAHS treated at the Affiliated Sixth People's Hospital Otolaryngology Department at Shanghai Jiao Tong University between March 2017 and May 2018 were included in the study. The control group consisted of 53 age‐ and sex‐matched healthy people with normal hearing, no snoring history, and AHI < 5. All participants underwent high‐resolution MRI and overnight polysomnography (PSG). At baseline, all participants completed the mini‐mental state examination (MMSE) and underwent a comprehensive interview to obtain information regarding age, sex, duration of snoring history, history of current and previous illnesses, and medical treatments. The interview questionnaire and PSG data were collected and analyzed by two independent investigators. Patients with a history of DM, hypertension, and atherosclerosis, defined as previous diagnosis according to standard clinical guidelines, and those with sleep disorders other than OSAHS or who had received treatment for OSAHS were excluded from the study. Additional exclusion criteria were congestive heart failure, intrinsic pulmonary disease, drug dependence, alcoholism, severe psychiatric disturbance, and pregnancy.

### 2.3 Evoked‐related potential (ERP) study

All patients underwent an evoked‐related potential (ERP) study. Electroencephalogram (EEG) data were recorded using a 256 channel HydroCel Geodesic Sensor Net (Electrical Geodesics, Inc.) with Cz as the reference channel. All electrode impedances were monitored and maintained below 50 kΩ. We used oddball stimuli with speech sounds using E‐Prime software (2.0; Psychology Software Tools Inc.). The auditory oddball task involved the presentation of a 1,000 Hz pure tone, with an oddball target stimulus presented at 2,000 Hz. The 1,000 Hz stimulus was presented in 85% of the trials, and the 2,000 Hz stimulus was presented in 15% of the trials. In total, the task consisted of 1,000 auditory stimuli at random inter‐stimulus intervals (ISI) ranging from 850 to 1,450 ms. The sound stimuli (75 dB) were delivered through two loudspeakers located 100 cm from the subjects. During the P3 cortical wave test, the subjects were instructed to close their eyes and press a button held in the right hand each time they detected a target tone.

### 2.4 MRI protocol and assessment

Participants underwent high‐resolution three‐dimensional MRI to detect silent markers of SVD, including rated VRS, white matter lesions, lacunar infarcts, and deep microbleeds according to the definition provided by STandards for ReportIng Vascular changes on nEuroimaging (STRIVE; Wardlaw et al., 2013). Brain MRI was performed using a Siemens 3 T Verio scanner (Magnetom; Siemens) using the same T1‐ and T2‐weighted and fluid‐attenuated inversion recovery (FLAIR) sequences and susceptibility‐ and diffusion‐weighted imaging. A neuroradiologist blinded to the clinical data reviewed all images independently and recorded the location and rated the VRS, white matter lesions, lacunar infarcts, and deep microbleeds. Recent small subcortical infarct was defined as neuroimaging evidence of recent infarction in the territory of one perforating arteriole with imaging features or clinical symptoms consistent with a lesion occurring in the previous few weeks. A lacune of presumed vascular origin was defined as a round or ovoid, subcortical, fluid‐filled cavity 3–15 mm in diameter consistent with a previous acute small subcortical infarct or hemorrhage in the territory of one perforating arteriole. A white matter lesion was defined as a signal abnormality of variable size in the white matter, that is, hyperintensity on T2‐weighted images such as fluid‐attenuated inversion recovery, without cavitation (Wardlaw et al., 2013). We defined VRS as <2 mm round or linear cerebrospinal fluid (CSF) isointense lesions along the course of penetrating arteries. Enlarged VRS were defined as round, ovoid, or linear CSF‐like signal lesions (hypointense on T1‐weighted and hyperintense on T2‐weighted images) with smooth delineated contours and maximum diameter >3 mm located in areas supplied by perforating arteries. VRS were rated on T2‐weighted images (with all sequences available) in the basal ganglia and centrum semiovale and graded as 0 = none, 1 = 1–10, 2 = 11–20, 3 = 21–40, and 4 = >40. Enlarged VRS were counted per side using the worse side where asymmetry existed (Doubal, MacLullich, Ferguson, Dennis, & Wardlaw, 2010). Total VRS scores were calculated as the sum of the basal ganglia and centrum semiovale VRS scores. We recorded the presence of white matter lesions, lacunar infarcts, and deep microbleeds.

### 2.5 Polysomnography

Polysomnography is the gold standard for the diagnosis of OSAHS. PSG (Alice 4 system; Respironics Inc.) recordings were scored manually according to standard criteria by the same experienced technician. Respiratory events were scored according to the American Academic Sleep Medicine criteria: Apnea was defined as complete cessation of airflow lasting for ≥10 s; hypopnea was defined as either a ≥50% reduction in airflow for ≥10 s, or a <50%, but discernible, reduction in airflow accompanied by a decrease in oxyhemoglobin saturation (SpO_2_) ≥4% or arousal. The apnea–hypopnea index (AHI) indicated the number of apnea and hypopnea events per hour during sleep, based on the results of the overnight PSG. Data without pulse SpO_2_ values, illegible recordings, and recordings <2 hr duration were excluded from analysis. OSAHS was rated as no OSAHS, AHI <5; mild, AHI 5 to <15; moderate, AHI 15 to <30; and severe, AHI ≥30 (Rodrigues, Dibbern, Goulart, & Palma, 2010). Subjects were divided into severe OSAHS (AHI ≥ 30/hr) and control (healthy people) groups.

### 2.6 Statistical analysis

Electroencephalogram was continuously recorded at a sampling rate of 250 Hz. The data were processed using Net Station (Electrical Geodesics, Inc.). The Statistical Package for the Social Sciences version 16 (SPSS Inc.) was used to conduct the statistical tests. We first assessed univariate associations of the total VRS scores followed by a multiple linear regression analysis to determine independent associations of the total VRS scores with vascular risk factors, lacunar stroke subtype, and MRI markers of cerebral SVD. Two‐sided Student's *t* test was also carried out to analyze differences of P3 amplitudes and demographic and sleep parameters, and then, chi‐square test was used to compare MRI findings between groups. A logistic regression analysis was performed to assess the association between silent cerebral SVD and the severity of OSAHS, while the association between silent cerebral SVD and cognitive impairment evaluated by P3 cortical wave test was also calculated.

## 3 RESULTS

The study included 72 patients with OSAHS (57 males and 15 females) aged 29–58 years, (mean, 47.5 ± 6.6 years) and 53 controls without OSAHS (39 males and 14 females) aged 32–58 years (mean, 46.4 ± 6.7 years). The duration of snoring history ranged from 5 to 22 years. The mean duration of snoring was 8.5 years, and the mean AHI value was 51.6 ± 12.1. The demographic and clinical characteristics of the participants are shown in Table 1.

**Table 1:** Characteristics of study subjects in OSAHS group and control group

Parameters	OSAHS group	Control group	*p* Value
Demographic			
*N* (%, male)	57 (79.2)	39 (73.6)	*p* = .154
Age (mean ± *SD*)	47.5 ± 6.6	46.4 ± 6.7	*p* = .981
BMI (kg/m^2^)	28.2 ± 2.3	22.8 ± 2.0	*p* = .000
MMSE (mean)	29.97	30	*p* = .22
Sleep parameters			
Snoring history (years)	8.5 ± 3.9	0	*p* = .000
AHI (mean ± *SD*)	51.6 ± 12.1	3.5 ± 1.1	*p* = .000
Lowest SpO_2_ duration sleep, %	65.34 ± 11.43	92.45 ± 4.56	*p* = .000
Mean SpO_2_ duration sleep, %	78.7 ± 6.1	94.3 ± 1.6	*p* = .000
AI (mean ± *SD*)	31.9 ± 6.2	14.5 ± 7.0	*p* = .000

Abbreviations: AHI, apnea–hypopnea index; AI, arousal index; BMI, body mass index; MMSE, mini‐mental state examination; OSAHS, obstructive sleep apnea–hypopnea syndrome; SpO_2_, percutaneous blood oxygen saturation.

**Figure 1:**
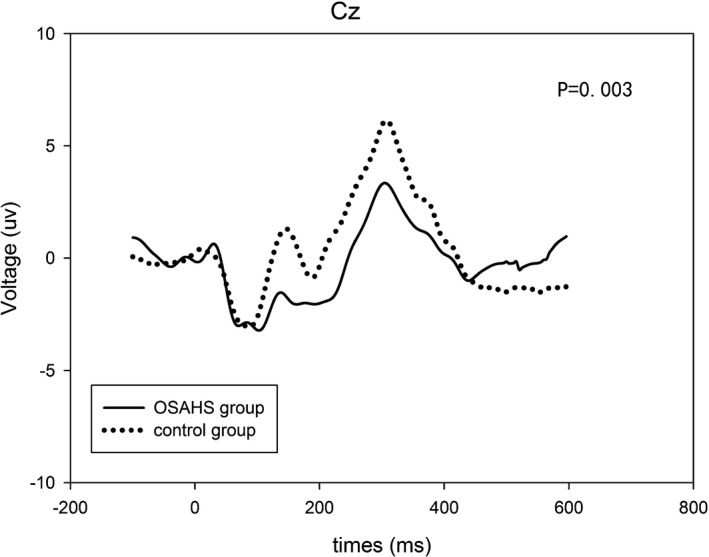
Grand average ERP waveforms for the standard and target stimuli in the OSAHS and control groups at Cz. Solid thick line: ERP for the target stimuli in OSAHS group, Dashed thick line: ERP for the target stimuli in control group

The MMSE scores of the patients with severe OSAHS were not significantly different from those of the control subjects. The ERP waveforms for the standard and target speech stimuli showing P3 components are shown in Figure 1. The mean P3 latency and amplitude were 308 ms and 6.14 μV, respectively, in the control group and 304 ms and 3.34 μV, respectively, in the OSAHS group. The P3 amplitudes of the patients with severe OSAHS were significantly smaller than those of the control subjects at Cz (*p* < .05), whereas the latency was not significantly different between groups.

### 3.1 MRI results

**Figure 2:**
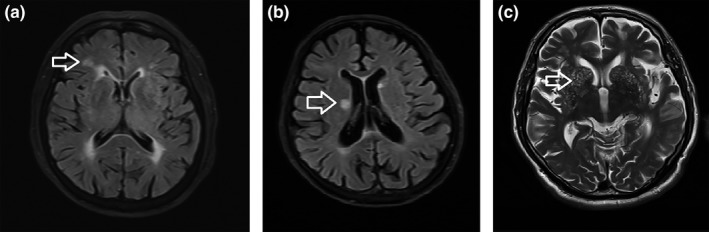
Representative MRI images of white matter abnormalities (a), lacune (b), and enlarged VRS (c) in different regions of the brain in one of the OSAHS patients

Enlarged VRS, white matter lesions, lacunar infarcts, and deep microbleeds were observed in the brains of patients with OSAHS. Deep microbleeds were identified and rated according to the Microbleed Anatomical Rating Scale (Figure 2). White matter lesions were observed in eight (11.1%) OSAHS and five (9.4%) control subjects; silent lacunar infarcts were found in four (5.6%) OSAHS and two (3.8%) control subjects; enlarged VRS were observed in 60 (83.3%) OSAHS and 16 (30.2%) control subjects; and no deep microbleeds were found in either group (Table 2). There were no significant differences in white matter lesions, lacunar infarcts, and deep microbleeds.

**Table 2:** Summary of MRI findings (enlarged VRS, WMH, lacunar infarcts, and deep microbleeds) in OSAHS and control groups

Demographics	OSAHS group	Control group	*p* Value
Number	72	53	
Enlarged VRS, *n* (%)	60 (83.3)	16 (30.2)	*p* < .001
Grade 1, *n* (%)	3 (5)	12 (75)	*p* < .001
Grade 2, *n* (%)	15 (25)	4 (25)	
Grade 3, *n* (%)	34 (57)	0 (0)	
Grade 4, *n* (%)	8 (13)	0 (0)	
WMH, *n* (%)	8 (11.1)	5 (9.4)	*p* < .001
Lacunar infarcts, *n* (%)	4 (5.6)	2 (3.8)	*p* < .001
Deep microbleeds, *n* (%)	0 (0)	0 (0)	

Abbreviations: MRI, magnetic resonance imaging; OSAHS, obstructive sleep apnea–hypopnea syndrome; VRS, Virchow–Robin spaces.

**Figure 3:**
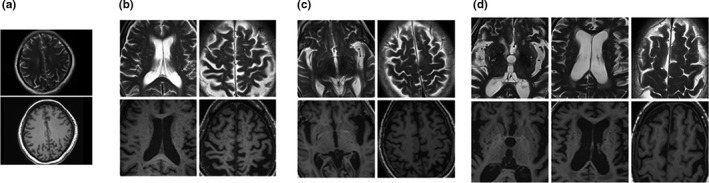
VRS were rated on T2‐weighted images (with all sequences available) in the basal ganglia and centrum semiovale and graded according to score as 1 = 1–10 (a), 2 = 11–20 (b), 3 = 21–40 (c), and 4 ≥ 40 (d). Locations are shown on T1‐weighted three‐dimensional images

**Figure 4:**
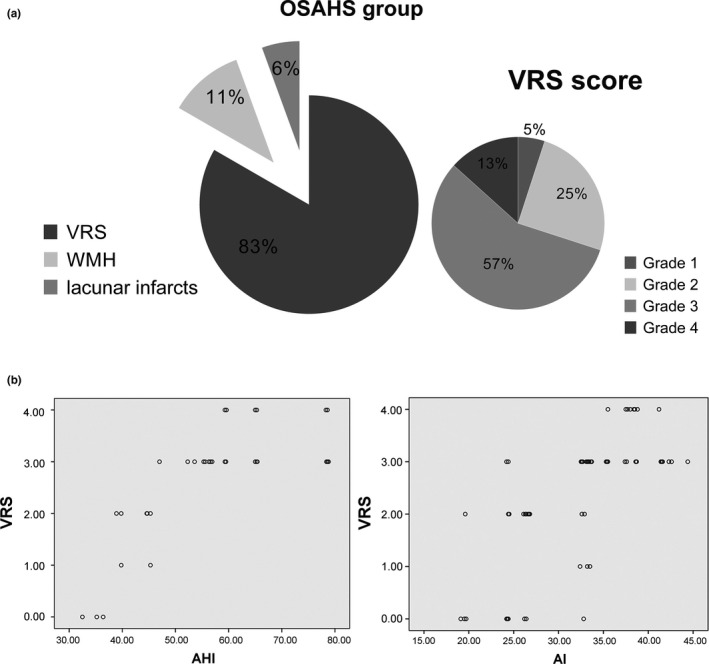
(a) The percentages of silent cerebral small vessel disease including rated VRS, white matter lesions, and lacunar infarcts in OSAHS subjects were displayed in the left part, and the percentages of VRS grades 1–4 OSAHS subjects were shown in the right part. (b) The distributions of VRS total score according to AHI and AI in OSAHS subjects were demonstrated

Enlarged VRS were most common in the bilateral basal ganglia regions (Figure 3) and significantly more prevalent in the OSAHS group. The percentage of patients with OSAHS in VRS grades 1–4 were 5%, 25%, 57%, and 13%, respectively (Figure 4a), compared with 75%, 25%, 0%, and 0%, respectively, in the control group. As presented in Figure 4b, the VRS total score was significantly positively correlated with the AHI (*r* = .63, *p* < .001) and AI (*r* = 0.55, *p* < .001) in the OSAHS group. Furthermore, the VRS total score was negatively correlated with the mean SpO_2_ (*r* = −.35, *p* = .003) in the OSAHS group, while the VRS total score was negatively correlated with P3 amplitudes (*r* = −.46, *p* < .001).

## 4 DISCUSSION

### 4.1 Synopsis of new findings

The present study demonstrated that silent cerebral SVD was more prevalent in patients with OSAHS than in the controls. Enlarged VRS were observed in the basal ganglia and centrum semiovale. The P3 amplitudes of patients with severe OSAHS were smaller than those of the control subjects, which is an indicator of cognitive impairment. VRS volume was positively correlated with AHI and AI values, and was negatively correlated with P3 amplitudes, suggesting that enlarged VRS may contribute to cognitive impairment.

### 4.2 Inferences with other studies

Mounting evidence suggests that OSAHS is associated with neurocognitive impairment (Chen et al., 2015). The auditory P3 is an electrophysiological event associated with attention processes in tasks that require target detection and involve sensory perception (Smart, Segalowitz, Mulligan, & MacDonald, 2014). Because P3 reflects a neuropsychobiological event, analysis of its parameters allows one to infer cognitive function in an objective fashion. The P3 amplitude reflects brain activity in the parietal–temporal and prefrontal areas associated with auditory memory, which is reduced in individuals with OSAHS. Previous studies have shown that P3 amplitude is significantly reduced in individuals with severe OSAHS, suggesting cognitive dysfunction. However, the mechanism remains unclear.

MRI‐visible VRS are potential neuroimaging markers of cerebral SVD, and several imaging markers of SVD have been independently associated with cognitive decline in a range of clinical cohorts (Hurford et al., 2014). Previously, SVD has been regarded as a slowly progressing disease that affects frontal‐subcortical networks, leading to corresponding frontal symptoms (Cummings, 1993). These symptoms were constituted by loss of mental processing speed, executive function, affected cognitive function, motor performance, and mood regulation. In our results, the P3 amplitudes of patients with severe OSAHS were smaller than those of the control subjects. VRS volume was negatively correlated with P3 amplitudes, suggesting that enlarged VRS may contribute to cognitive impairment.

However, there is little evidence to support an association between OSAHS and cerebral SVD. VRS detected by MRI have been associated with age‐related disorders and vascular risk factors (Zhang et al., 2014). We found no significant differences in age or sex between the OSAHS and control groups, and patients with a history of DM, hypertension, and atherosclerosis were excluded from our study. Our finding of enlarged VRS in the basal ganglia and centrum semiovale was consistent with that of a previous study (Del Brutto et al., 2015). Moreover, we found that AHI and AI values were positively correlated with the VRS score, particularly in the basal ganglia. VRS volume was positively correlated with higher AHI and AI values, suggesting that recurrent hypoxic episodes during sleep and sleep fragmentation may be associated with inefficient perivascular drainage, potentially leading to enlargement of the perivascular space. Moreover, the positive correlation between VRS and AI provides further support for VRS as a marker of suboptimal perivascular clearance during sleep disturbance.

### 4.3 Clinical strengths of the study

Our finding of a negative correlation between VRS and mean oxygen saturation during sleep suggests that intermittent hypoxia may be an important contributing factor for cognitive dysfunction in OSAHS. Enlarged VRS are increasingly recognized as a marker of disease rather than simply a marker of aging; however, few studies have investigated the association between OSAHS and VRS. Our study provides further evidence that the P3 amplitudes of patients with severe OSAHS were smaller than those of the control subjects, and enlarged VRS volume negatively correlated with P3 amplitudes may contribute to cognitive impairment.

### 4.4 Our limitations

The main limitation of our study was its retrospective design, which restricted our analysis to available, routinely collected data. Secondly, we did not control for BMI in patients and control group, while high BMI is significantly related to the occurrence of OSA; thus, we consider the significant difference of BMI as inherent characteristics between groups. Additionally, PSG measurements obtained in the hospital may not provide a true representation of the normal sleep patterns exhibited at home in a more natural setting. A technological limitation of our study was the lack of a noninvasive reliable method for discriminating between arteries and veins in vivo using MRI. The strengths of our study include the use of high‐resolution MRI and our definition of enlarged VRS based on strict anatomical and imaging criteria, and the use of a highly reliable assessment method.

## 5 CONCLUSIONS

In summary, silent cerebral SVD was more prevalent in OSAHS patients. Neuroimaging consequences of cerebral small vessel disease were particularly found in the basal ganglia and centrum semiovale. The enlarged VRS and decreased P3 amplitudes may be a useful imaging biomarker of cognitive impairment in patients with obstructive sleep apnea–hypopnea syndrome.

## CONFLICT OF INTEREST

The authors declare that there is no conflict of interest statement.

## Data Availability

The data that support the findings of this study are available from the corresponding author upon reasonable request.
